# The Effects of Sage (*Salvia officinalis* L.) Leaf Powder Supplementation in Broiler Diets on Performance, Gut Health, and Meat Quality

**DOI:** 10.3390/vetsci12121148

**Published:** 2025-12-02

**Authors:** Hüseyin Çayan, İsa Coşkun

**Affiliations:** Department of Animal Science, Faculty of Agriculture, Kirsehir Ahi Evran University, Kirsehir 40100, Türkiye; isa.coskun@ahievran.edu.tr

**Keywords:** sage, broiler, meat color, MDA, gut health

## Abstract

This study was conducted to evaluate the effects of dietary supplementation with different levels of sage (*Salvia officinalis* L.) leaf powder on growth performance, internal organ development, cecal microbiota, intestinal histomorphology, meat quality characteristics, and breast meat malondialdehyde (MDA) levels in broiler chickens. A total of 160 one-day-old male Ross 308 broiler chicks of uniform health status and body weight were randomly allocated to four treatment groups, each consisting of four replicates with 10 birds per replicate. The experimental diets were formulated by supplementing the basal diet with 0, 2, 4, or 8 g/kg of sage leaf powder. The trial was conducted for 21 days in four-tier battery cages under controlled environmental conditions, with feed and water supplied ad libitum. At the end of the experiment, dietary inclusion of sage leaf powder at 2 g/kg significantly improved daily body weight gain (*p* < 0.05), while feed intake and feed conversion ratio were not affected (*p* > 0.05). No significant differences were detected among the treatment groups in carcass traits, meat pH, or color parameters. However, marked improvements were observed in intestinal histomorphology. In addition, breast meat samples from birds receiving sage-supplemented diets displayed slightly lower MDA concentrations during storage compared to the control group. In conclusion, dietary supplementation with sage leaf powder improved growth performance, enhanced intestinal health, and demonstrated potential as a natural feed additive for broiler nutrition; however, it did not exert a statistically significant effect on lipid oxidation under the conditions of this study.

## Simple Summary

The use of antibiotics as growth promoters in poultry production has been prohibited worldwide due to their adverse effects on public health and the growing concern about antibiotic resistance. Consequently, considerable attention has been directed toward identifying safe and natural feed additives that can maintain animal health and productivity. Sage (*Salvia officinalis*), a widely recognized medicinal and aromatic plant, contains bioactive compounds with antibacterial, antioxidant, and anti-inflammatory properties. The present study examined whether dietary supplementation with sage leaf powder could naturally enhance growth performance, digestion, gut health, and meat quality in broiler chickens. The results indicated that broiler chicks receiving dietary sage exhibited improved growth performance along with a healthier intestinal structure and function. During the meat storage period, oxidative deterioration values were marginally lower in the sage-supplemented groups compared to the control group, although these differences were slight. These findings suggest that sage leaf powder has the potential to serve as a natural alternative to antibiotics in poultry diets. Incorporating sage into animal nutrition may contribute to safer and more sustainable poultry production systems and better meet the preferences of consumers who demand antibiotic-free animal products.

## 1. Introduction

Poultry production has become one of the fastest growing and most technologically advanced sectors of animal agriculture worldwide. Due to its short production cycle, low production costs, and the high nutritional value of poultry products (meat and eggs), this sector plays a strategic role in meeting the rising global demand for animal-derived protein driven by rapid population growth. Broiler chickens possess an exceptional capacity to help close the global protein gap, owing to their rapid growth rates, efficient feed conversion, and the favorable nutritional profile of their meat. As a result, poultry production represents a critical component of sustainable food security and economic development in both developed and developing nations [1,2].

In broiler production, the primary objective is to achieve rapid body weight gain and efficient feed utilization within the shortest possible time. For many years, antibiotics were widely used as growth-promoting feed additives to support these goals. However, the excessive and non-therapeutic use of antibiotics has led to the emergence of antibiotic-resistant bacterial strains and the accumulation of drug residues in animal-derived foods, both of which pose serious risks to public health [3]. The ban imposed by the European Union on the use of antibiotics as growth-promoting agents in animal feed has stimulated extensive research efforts to identify natural, safe, and residue-free alternatives capable of reproducing the beneficial effects of antibiotics [4].

Within this context, natural feed additives such as probiotics, prebiotics, organic acids, enzymes, and medicinal and aromatic plants have gained increasing attention in poultry nutrition [5,6]. Medicinal and aromatic plants are regarded as promising natural growth promoters due to their antimicrobial, antioxidant, anti-inflammatory, antistress, and digestion-enhancing properties [7]. Among these plants, sage is particularly noteworthy for its strong antioxidant and antimicrobial activities, which are largely attributed to its essential oil constituents—such as thujone, cineole, and borneol—as well as its flavonoid derivatives [8,9].

Previous studies investigating the use of sage in poultry diets have primarily focused on its essential oils, extracts, or combinations with other herbal additives. Several reports have shown that the inclusion of sage or mixed essential oils in broiler diets can improve feed efficiency and carcass yield [10,11], reduce lipid oxidation in meat and eggs [3], and enhance immune responses [10]. In contrast, other studies have reported no significant effects on growth performance or intestinal morphology [9,12], suggesting that the observed outcomes may vary depending on the form, dosage, and method of sage application in poultry diets.

In light of this background, the objective of the present study was to evaluate the effects of dietary supplementation with sage leaf powder on growth performance, meat quality characteristics, MDA formation during refrigerated storage, and intestinal health in broiler chickens.

## 2. Materials and Methods

### 2.1. Animals and Experimental Design

In this study, 160 healthy one-day-old male Ross 308 broiler chicks with an average initial body weight of 40.7 g were used. Chicks of similar body weight were randomly assigned to four dietary treatments, each consisting of four replicates with 10 chicks per replicate. The dietary treatments included a control group without sage supplementation (CON) and three treatment groups supplemented with 2, 4, or 8 g/kg of sage leaf powder (S2, S4, and S8, respectively). The trial lasted 21 days and was conducted in broiler rearing cages. Throughout the experimental period, all chicks were fed a starter diet containing 21.5% crude protein and 3100 kcal/kg metabolizable energy (Table 1).

The sage used as a feed additive in this study was obtained in dried form from the Agricultural Practice and Research Center of Kırşehir Ahi Evran University. In the experiment, the standard compound feeds used as basal diets were supplemented solely with different levels of sage leaf powder, and no additional additives were included. The ingredients and nutrient composition of the basal diet are presented in Table 1. The nutrient composition of sage leaves was analyzed at the Central Research and Application Laboratory of Kırşehir Ahi Evran University, and the corresponding values are (on DM basis): dry matter 92.00%, crude protein 22.80%, ether extract 14.31%, crude ash 9.92%, starch 15.84%, acid detergent fiber (ADF) 21.35%, and neutral detergent fiber (NDF) 23.95%.

To evaluate the antioxidant capacity of sage (*Salvia officinalis* L.), the DPPH free radical scavenging activity was determined according to the method described by [13]. The concentration of the extract or standard compound required to inhibit 50% of the DPPH radical (IC_50_) was calculated and used as an indicator of antioxidant capacity. Based on the percentage inhibition values obtained at different concentrations, the IC_50_ of the sage extract was determined and is presented in Table 2. Since the IC_50_ value is inversely related to antioxidant activity, a lower IC_50_ indicates a stronger antioxidant effect. The IC_50_ value of the sage extract was found to be lower than that of the standard antioxidant BHT, demonstrating that the sage extract exhibited higher antioxidant capacity.

The chicks were housed under controlled environmental conditions in a four-tier battery cage system consisting of four blocks and a total of 16 stainless-steel cages, each measuring 92 × 33 × 45 cm. Feed and water were supplied ad libitum, and a 24 h lighting schedule was implemented throughout the experiment. To minimize positional effects, equal numbers of chicks were allocated to the upper and lower tiers. During the first 10 days, two plastic feeders (1 kg capacity) were placed in each cage, after which feed troughs were installed at the front of each compartment. Drinking water was provided via nipple drinkers positioned on each tier. The room temperature was maintained at 33 ± 1 °C during the first week and gradually reduced to 24 ± 1 °C, while relative humidity was kept above 60%.

### 2.2. Determination of Performance Parameters

Body weight measurements were taken on days 7, 14, and 21 of the experiment. Feed intake (FI) was determined by recording the amount of feed offered and subtracting the residual feed. Feed conversion ratio (FCR) was calculated as FCR = FI ÷ BWG. Throughout the trial, all mortalities within each group were recorded, and the mortality rate (%) was calculated at the end of the experimental period.

### 2.3. Determination of Slaughter and Carcass Characteristics

At the end of the experiment (day 21), all birds were weighed to determine their final body weights. From each treatment group, eight birds (two chicks closest to the replicate mean body weight from each repetition), totaling 32 birds, were selected for slaughter and subsequent analyses. After slaughter, the length of the digestive tract was measured, and the weights of the heart, gizzard (with the lining removed), liver, proventriculus, and bursa of Fabricius were recorded using a precision balance with an accuracy of 0.01 g.

Post-slaughter, pH measurements were taken from the breast and thigh muscles of each eviscerated and cleaned carcass. The pH values of both muscles were recorded at three different points on the left side of the carcass using a digital pH meter (Testo 205) equipped with a solid-state electrode.

Color measurements of the breast and thigh muscles were performed on the skinless surface of the same anatomical regions where pH was measured, using a Minolta CR-410 Chroma Meter (Minolta Camera Co., Osaka, Japan) calibrated with a white standard plate (Minolta calibration plate No. 21733001, Y = 92.6, x = 0.3136, y = 0.3196). For each sample, color values were recorded at three different points.

### 2.4. Determination of Histomorphological Characteristics of the Small Intestine

On day 21 of the study, duodenal, jejunal, and ileal samples collected from slaughtered birds in each treatment group were fixed in 10% formaldehyde. For histological analysis, the tissues were embedded in paraffin, sectioned at a thickness of 5 μm, and mounted onto glass slides. The slides were then deparaffinized in xylene, passed through a graded alcohol series, and cleared to remove residual xylene. The prepared tissue sections were stained with hematoxylin and eosin (H&E) and photographed using an AxioCam ERc 5s 5-megapixel digital microscope camera (ZEISS Primo Star, Jena, Germany). Villus height and crypt depth were measured from the microphotographs of each sample using ZEN 2012 SP2 image processing and analysis software [14].

### 2.5. Determination of Meat Shelf Life

Lipid oxidation in the meat samples was evaluated using the 2-thiobarbituric acid reactive substances (TBARS) method. Breast meat samples (10 g) were collected from two chicks per replicate, yielding a total of 32 samples (eight per treatment group). Each sample was placed in an airtight plastic bag to prevent exposure to air and stored at 4 °C until analysis. MDA measurements were performed on days 3, 5, and 7 of storage.

For analysis, each sample was combined with 50 mL of distilled water preheated to 50 °C and homogenized for 2 min using an Ultra-Turrax homogenizer (IKA T18). The homogenate was then transferred into distillation tubes, followed by the addition of 47.5 mL of distilled water, 2.5 mL of 4 N HCl, and a small amount of paraffin to reduce foaming. Boiling stones were added to ensure smooth boiling during distillation. The mixture was distilled under low steam intensity until 50 mL of distillate was collected.

From the distillate, 5 mL was pipetted into glass tubes and mixed with 5 mL of thiobarbituric acid (TBA) reagent. Blank samples were prepared by replacing the distillate with 5 mL of distilled water. After vortexing, all tubes were heated in a boiling water bath for 35 min and then rapidly cooled under running water for 10 min. Absorbance was measured at 538 nm using a spectrophotometer (Shimadzu UVmini-1240, Shimadzu Corporation, Tokyo, Japan). TBARS values were calculated by multiplying the absorbance by 7.8 and expressed as mg malondialdehyde (MDA) per kg of sample [15].

### 2.6. Determination of Cecal Microbiota

For the determination of cecal microbiota, cecal content samples (1 g) were collected from two chicks per replicate, resulting in a total of 32 samples (eight per treatment group). Each sample was mixed with 9 mL of peptone water and homogenized using a vortex mixer to achieve a uniform suspension. Serial tenfold dilutions (1:10) were then prepared from the initial mixture. Microbial enumeration was performed using selective culture media: *Lactobacillus* spp. were plated on MRS agar (De Man–Rogosa–Sharpe, MERCK 1.10660) and incubated at 37 °C for three days; yeasts were cultured on Malt Extract Agar (MEA, MERCK 1.05398) and incubated at 30 °C for three days; and *Escherichia coli* were grown on Eosin Methylene Blue (EMB) agar (MERCK 101347) and incubated at 37 °C for three days. After incubation, colonies were counted and expressed as log_10_ colony-forming units (CFU) per gram of cecal content.

### 2.7. Statistical Analysis

The data obtained from the experiment were analyzed using one-way analysis of variance (ANOVA) under a completely randomized design. Orthogonal polynomial contrast coefficients were applied to assess the linear and quadratic effects of increasing sage inclusion levels when significant treatment effects were detected. Differences among group means were determined using Duncan’s multiple range test, and statistically significant differences between treatments are denoted by the letters a, b, and c in all tables. All statistical analyses were conducted using the SPSS 21.0 for Windows Evaluation Version software package.

## 3. Results

The findings of the study examining the effects of different levels of sage leaf powder supplementation in broiler diets on performance, slaughter and carcass characteristics, internal organ parameters, intestinal histomorphology, meat shelf life, and cecal microbiota are presented below.

### 3.1. Performance Parameters

According to Table 3, supplementation of broiler diets with 2 g/kg sage leaf powder (S2) significantly increased daily and total body weight gain (DBWG and TBWG) compared to the control group (*p* < 0.05). However, no significant differences were observed among the groups in feed intake (FI), feed conversion ratio (FCR), or survivability rate (*p* > 0.05). A quadratic response was detected for growth performance, wherein the 2 g/kg supplementation level improved performance, but this beneficial effect diminished as the sage inclusion level increased.

### 3.2. Slaughter, Carcass, and Meat Quality Characteristics

At the end of the 21-day feeding trial, the findings regarding slaughter and carcass characteristics of broiler chickens fed diets containing different levels of sage leaf powder are presented in Table 4, while the effects on meat quality parameters are shown in Table 5.

According to Table 4, supplementation of broiler diets with different levels of sage leaf powder (2, 4, and 8 g/kg) did not significantly affect most slaughter and internal organ parameters (*p* > 0.05), except for gizzard weight, which differed significantly among treatments. Gizzard weight decreased linearly as the level of sage supplementation increased (*p* = 0.007).

According to Table 5, supplementation of broiler diets with different levels of sage leaf powder (2, 4, and 8 g/kg) did not significantly influence the color parameters (L*, a*, b*) or pH values of either breast or thigh meat (*p* > 0.05). Although the differences were not statistically significant, the a* values of both thigh and breast meat exhibited a linear decreasing trend with increasing levels of sage supplementation.

### 3.3. Small Intestinal Histomorphology

The effects of sage leaf supplementation to broiler diet on small intestinal histomorphology are presented in Table 6.

As shown in Table 6, the inclusion of sage leaf powder in broiler diets significantly affected various intestinal histological parameters (*p* < 0.05). While villus length in the duodenum was not influenced by the treatments, crypt depth and the villus height-to-crypt depth (VL/CD) ratio were significantly altered. The most favorable crypt depth values in the duodenum were observed in the group supplemented with 4 g/kg sage leaf powder.

In both the jejunum and ileum, all evaluated histomorphological parameters were significantly affected by the dietary treatments (*p* < 0.05). The highest villus lengths in these intestinal segments were found in birds fed diets containing 2 g/kg sage leaf powder. Notably, this group also exhibited greater body weight gain, suggesting a positive association between villus development and growth performance. Increased villus height reflects an expanded intestinal absorptive surface area, which can enhance nutrient uptake efficiency and overall feed utilization.

As shown in Table 6, the inclusion of sage leaf powder in broiler diets significantly affected the intestinal histological structure (*p* < 0.05). While villus length in the duodenum was not influenced by the treatments, crypt depth and the villus height-to-crypt depth (VL/CD) ratio were significantly affected. The best values for crypt depth in the duodenum were observed in the group supplemented with 4 g/kg sage leaf powder.

In both the jejunum and ileum, all examined histomorphological parameters were significantly influenced by dietary treatments (*p* < 0.05). The highest villus lengths in these intestinal segments were obtained from birds fed diets containing 2 g/kg sage leaf powder. Moreover, these groups also showed higher body weight gains, indicating a positive relationship between villus development and growth performance. The increased villus height suggests an expansion of the intestinal surface area, which enhances the efficiency of nutrient absorption and overall feed utilization.

### 3.4. Cecal Microbiota

The effects of sage leaf supplementation into broiler on cecal microbiota are presented in Table 7. As shown in the table, the inclusion of sage leaf powder in broiler diets had no statistically significant effect on the populations of *E. coli* and Lactic acid bacteria (*Lactobacillus* spp.) (*p* > 0.05). However, the number of *Enterococcus* spp. bacteria was significantly affected by the dietary treatments (*p* < 0.05). A clear linear decrease in *Enterococcus* spp. counts was observed with increasing levels of sage leaf powder supplementation, indicating that higher sage inclusion levels exerted a suppressive effect on this bacterial group.

### 3.5. Meat Shelf Life

At the end of the 21-day feeding trial, breast meat samples collected from broilers fed diets supplemented with different levels of sage leaf powder were analyzed to evaluate meat shelf life. The malondialdehyde (MDA) values, which reflect lipid oxidation levels at different storage times, are presented in Table 8. As shown in Table 8, sage leaf powder—despite its known antioxidant properties—did not exert a statistically significant effect on breast meat shelf life at any of the storage times evaluated (*p* > 0.05).

## 4. Discussion

This study was conducted to evaluate the effects of dietary supplementation with sage (*Salvia officinalis* L.) leaves on growth performance, internal organ development, cecal microbiota, intestinal histomorphology, meat characteristics, and the malondialdehyde (MDA) content of breast meat in broiler chickens. The results demonstrated that dietary inclusion of sage significantly affected body weight gain (BWG) (*p* < 0.05). The highest BWG value (31.51 g) was recorded in the S2 group (2 g/kg), indicating that low-level supplementation of sage positively contributed to growth performance. However, increasing the supplementation levels (S4 and S8) resulted in a slight decrease in BWG compared with the S2 group, although both levels still produced higher BWG values than the control. Sage supplementation did not have a statistically significant effect on feed conversion ratio (FCR), yet a numerical improvement in FCR was observed in all treatment groups relative to the control. The improvements observed in growth parameters may be attributed to the antimicrobial and antioxidant properties of the secondary metabolites present in sage. These bioactive compounds may enhance intestinal health and stimulate digestive enzyme activity, thereby improving nutrient digestion and utilization and ultimately supporting better growth performance [16]. In line with these findings, Akdağ and Bozbağ [17] reported that supplementation with sage essential oil increased viability and body weight at 21 days of age and reduced total oxidant status, indicating a mitigation of heat stress–induced oxidative stress in broilers exposed to prolonged high ambient temperatures.

Previous studies evaluating medicinal and aromatic plants or their extracts as natural alternatives to antibiotic growth promoters have shown variable results. While some researchers have reported improvements in growth performance, feed conversion ratio (FCR), and feed cost efficiency [18,19,20], others have found no statistically significant differences compared with control groups [21,22]. The slight reduction in body weight gain observed at higher supplementation levels in the present study may be associated with the increased cellulose content of the diet, which could have negatively influenced growth. The sage leaves used in this experiment contained approximately 14% cellulose, resulting in higher cellulose intake in the treatment groups compared with the control. Similarly, Tejeda and Kim [23] noted that both the type and level of dietary fiber in poultry rations can negatively affect performance by altering intestinal development and limiting nutrient digestion.

In this study, no significant differences were observed among the groups with respect to digestive tract length or the weights of the heart, liver, proventriculus, bursa of Fabricius, and other edible internal organs. These findings for digestive tract length, heart, and proventriculus align with previous reports. Pisevo et al. [24] similarly found that sage extract had no effect on internal organ weights in broiler chickens. Comparable results have been reported in studies using other medicinal and aromatic plants: Kheiri et al. [25] and Coskun et al. [14] in quails, and Toghyani et al. [26], Adam et al. [27], and Vlaicu et al. [4] in broilers noted that dietary inclusion of thyme did not significantly influence internal organ development.

In contrast to these parameters, gizzard weight in the present study decreased significantly as sage supplementation levels increased (*p* < 0.05). This reduction may be associated with improved digestive efficiency. Bioactive compounds present in sage—such as cineole, rosmarinic acid, and carnosic acid—are believed to stimulate digestive enzyme secretion, thereby reducing the mechanical workload required by the gizzard during feed breakdown. As the muscular effort needed for grinding decreases, a corresponding reduction in gizzard weight may occur. Additionally, volatile constituents of *Lamiaceae* species, including sage, are known to exert antispasmodic effects on smooth muscle, potentially accelerating gastric–gizzard transit and further reducing mechanical strain. These antispasmodic and gastroprotective actions, along with the general smooth muscle–relaxing effects of essential oils, have been well documented in the pharmacological literature [28].

Meat quality is a multifactorial trait influenced by numerous biological and environmental factors. Carcass yield, visual appearance, and sensory attributes are of great importance to both consumers and the poultry industry. Among these attributes, color parameters—lightness (L*), redness (a*), and yellowness (b*)—play a critical role in shaping consumer preferences. Stress factors in poultry are known to accelerate glycolysis [29], which can increase the L* value of breast meat, reduce water-holding capacity, and ultimately impair overall meat quality [29]. In the present study, the inclusion of sage leaf powder did not significantly affect the pH or the L*, a*, and b* color parameters of broiler breast or thigh muscles (*p* > 0.05). These findings align with those reported by Akdağ and Bozbay [17], who similarly observed no significant changes in meat color in response to dietary sage essential oil. From a consumer standpoint, the absence of undesirable changes in meat color is favorable, as color is a key quality characteristic that strongly influences purchasing decisions and first impressions at the point of sale.

The inclusion of different levels of sage leaf powder in broiler diets significantly influenced the histological structure of the small intestine. Although villus height in the duodenum was not affected by the dietary treatments, crypt depth and the villus height-to-crypt depth (VH/CD) ratio differed significantly among the groups (*p* < 0.05). The most favorable duodenal crypt depth values were observed in birds supplemented with 4 g/kg of sage leaf powder. In both the jejunum and ileum, all evaluated histomorphological parameters were significantly affected by the dietary treatments (*p* < 0.05). The highest villus heights in these intestinal segments were recorded in the groups receiving 2 g/kg of sage supplementation. Consistent with this finding, these groups also exhibited greater body weight gain, suggesting that increased villus height may have enhanced nutrient absorption efficiency.

Nutrients and functional feed additives can influence the structural characteristics of the small intestine. Among the morphological indicators, villus height and crypt depth are widely recognized as essential parameters for evaluating digestive health and mucosal absorptive capacity. Increased villus height is generally associated with improved nutrient absorption efficiency, as taller villi provide a greater absorptive surface area [30]. Longer villi also slow the passage rate of digesta through the intestine, thereby enhancing moisture retention and feed conversion efficiency. Additionally, the villus height-to-crypt depth (VH/CD) ratio is a key indicator of intestinal digestive capacity, with higher ratios reflecting more efficient digestion and absorption processes [31].

In the present study, low to moderate supplementation levels of sage leaf powder (2–4 g/kg) improved villus height and expanded the absorptive surface area, whereas the highest level (8 g/kg) increased crypt depth, suggesting accelerated mucosal turnover and a disturbance in proportional balance. These morphological improvements in intestinal architecture indicate a more efficient utilization of dietary nutrients. Similarly, Farhadi et al. [10] reported beneficial effects of dietary sage supplementation on ileal histomorphology in broilers. Comparable findings have also been documented in studies involving other medicinal and aromatic plants [14,32,33,34], in which significant enhancements in small intestinal morphology were observed. The positive alterations in intestinal histomorphology observed in the current study may be attributed to the phenolic compounds (e.g., rosmarinic acid, carnosic acid) and volatile constituents (e.g., cineole, thujone) present in sage, which likely contributed to maintaining mucosal integrity through their antimicrobial, anti-inflammatory, and microbiota-modulating properties.

The intestinal microbial population is a major determinant of health and productivity in poultry. Broiler chicks, in particular, are highly susceptible to pathogenic microorganisms such as *Escherichia coli* and *Salmonella* during the early stages of life. Although pathogenic bacteria compete with the host for nutrients within the small intestine, the qualitative and quantitative composition of the intestinal microbiota can be influenced by several factors—including environmental conditions, housing system, microclimate, and diet composition—which may predispose birds to necrotic lesions in the intestinal mucosa [35,36].

In the present study, the inclusion of sage (*Salvia officinalis* L.) leaves in broiler diets exhibited a bacteriostatic effect, significantly reducing the cecal colony-forming units (CFU) of *Enterococcus* spp. (*p* < 0.05) and numerically lowering the CFU of *Escherichia coli*. Additionally, a numerical increase was observed in beneficial bacterial populations such as *Lactobacillus* spp. The reduction in harmful bacteria and the increase in *Lactobacillus* spp. counts observed in this study are consistent with the findings of Rasouli et al. [9], who reported similar trends with sage extract. These results also agree with studies showing decreased *E. coli* counts and increased *Lactobacillus* spp. populations in broilers receiving diets supplemented with various medicinal and aromatic plants [37,38,39,40].

The antimicrobial activity of sage has been attributed to its major bioactive constituents, including 1,8-cineole, thujone, and camphor [41,42]. The improvements observed in gut health in the present study may be explained by the antimicrobial, prebiotic, and antioxidant properties of these phenolic compounds and essential oils. Such properties likely contribute to maintaining a balanced intestinal microbiota by suppressing pathogenic bacteria and promoting the proliferation of beneficial microorganisms, thereby supporting intestinal integrity and overall poultry performance.

In the present study, dietary supplementation with sage leaf powder did not produce a statistically significant effect on lipid oxidation in broiler breast meat during refrigerated storage (*p* > 0.05). Although the sage-supplemented groups exhibited slightly lower MDA values compared to the control group, these numerical differences were minor and not biologically meaningful (Table 8). Therefore, they should not be interpreted as improvements in oxidative stability or shelf life. While several previous studies have reported reductions in lipid oxidation when sage extracts [10] or other phytogenic additives [4,10,14,43,44] were used, the absence of a significant effect in the present study may be attributed to the form and dosage of sage tested. Sage leaf powder contains lower concentrations of essential oils than extracts, and its antioxidant compounds may not have accumulated in muscle tissue at levels sufficient to influence lipid peroxidation. Additionally, the basal diet and storage conditions may have already created a relatively stable oxidative environment, thereby reducing the sensitivity to detect treatment-related differences.

Furthermore, color measurements of both breast and thigh meat revealed no meaningful differences among the groups. Because changes in meat color are closely linked to oxidative processes [45], the absence of color alterations supports the MDA findings and indicates that dietary sage did not substantially influence the oxidative stability of meat under the conditions of this study. Overall, although sage contains well-known antioxidant phenolic compounds, the levels of sage leaf powder tested in this experiment were insufficient to modify lipid oxidation or meat quality parameters associated with oxidative deterioration.

## 5. Conclusions

The findings of this study indicate that *Salvia officinalis* can serve as a natural phytogenic feed additive to support gut health, antioxidant status, and certain meat quality traits in broiler chickens. However, increasing the dosage of sage did not further enhance performance, likely due to the associated rise in dietary fiber content at higher inclusion levels. Future research should focus on determining the optimal supplementation level, evaluating preparations with higher essential oil concentrations, and exploring potential synergistic effects when combined with other phytobiotics or probiotics. Such investigations will help clarify the full potential of sage as a sustainable alternative to synthetic growth promoters in poultry nutrition.

## Figures and Tables

**Table 1:** Ingredients and nutrients composition of basal-diet fed to broiler chickens.

Ingredient	%
Corn	54.46
Soybean meal (%44 CP)	36.68
Vegetable oil	4.94
DL Methionine	0.30
NaCl	0.37
Limestone	0.84
Dicalcium phosphate	1.8
Mineral premix ^1^	0.15
Vitamin premix ^2^	0.20
L-Lysine	0.30
L-Threonine	0.05
**Calculated nutrients, as fed-basis**	
Crude protein (%)	21.50
ME (kcal/kg)	3100
Crude fiber (%)	3.686
Crude fat (%)	7.519
Crude ash (%)	5.684
Calcium	0.870
Available phosphorous	0.435

^1^ Each 1 kg of mineral premix contains: Mn, 120,000 mg; Fe, 60,000 mg; Zn, 100,000 mg; Cu, 16,000 mg; Co, 500 mg; I, 2000 mg; Se, 300 mg; Mo, 300 mg. ^2^ Each 1 kg of vitamin premix contains: Vit A, 12,500,000 IU; Vit D_3_, 5,000,000 IU; Vit E, 100,000 mg; Vit K_3_, 4000 mg; Vit B_1_, 3000 mg; Vit B_2_, 8000 mg; Niacin, 70,000 mg; Ca-D-Pantothenate, 20,000 mg; Vit B_6_, 5000 mg; Vit B_12_, 20 mg; D-Biotin, 200 mg; Folic acid, 2000 mg; Vit C, 100,000 mg.

**Table 2:** IC_50_, CUPRAC, and FRAP values of sage extract and BHT.

Parameters	DPPH IC_50_ (µg/mL)	CUPRAC	FRAP
Sage	121.95 ± 2.29	0.67	0.5
BHT	132.04 ± 10.44	1.46	1.25

DPPH: 1,1-Diphenyl-2-picrylhydrazyl; BHT: 2,6-di-tert-butyl-1-hydroxytoluene; IC_50_: concentration providing 50% inhibition of the radical; CUPRAC: CUPRAC activity expressed as Trolox equivalent antioxidant capacity (TEAC); and FRAP: FRAP activity expressed as Trolox equivalent antioxidant capacity (TEAC).

**Table 3:** Growth parameters of broilers (0–21 d) with dietary supplementation sage leaf powder.

Parameters	Sage Leaf, g/kg				SEM	*p*-Value		
	0	S2	S4	S8		ANOVA	L	Q
DBWG	29.32 ^b^	31.51 ^a^	30.43 ^a,b^	30.17 ^a,b^	0.28	0.03	0.381	0.005
DFI	43.96	45.65	43.62	44.11	0.40	0.31	0.661	0.453
FCR	1.50	1.45	1.43	1.46	0.02	0.55	0.153	0.593
SR%	95	100	97.5	92.5	1.55	0.38	0.347	0.049

Values represent means of four replicate pens with 40 birds from each treatment group; ^a,b^, There is a statistical difference between the averages shown with different letters (*p* < 0.05). SEM: Standard error of means, DBWG: Daily body weight gain, DFI: Daily feed intake, FCR Feed conversion ratio, SR: Survival rate, L: Linear, Q: quadratic. ANOVA: analysis of variance.

**Table 4:** Effect of sage leaf supplementation on slaughter and internal organ weights (cm-g/100 g live body weight).

Parameters	Sage Leaf, g/kg				SEM	*p*-Value		
	0	S2	S4	S8		ANOVA	L	Q
Gut length	21.74	22.56	23.03	21.71	0.26	0.198	0.642	0.099
Gizzard	3.35 ^a^	3.28 ^a,b^	3.06 ^a,b^	2.99 ^b^	0.05	0.048	0.007	0.997
Heart	0.72	0.77	0.74	0.76	0.01	0.612	0.408	0.563
Liver	2.45	2.54	2.49	2.54	0.04	0.778	0.517	0.748
Pro	0.64	0.64	0.60	0.61	0.01	0.408	0.172	0.944
Bursa	0.35	0.37	0.39	0.34	0.01	0.617	0.899	0.220
EIO	6.51	6.59	6.29	6.29	0.07	0.286	0.121	0.763

Values represent means of four replicate pens with 8 birds from each treatment; ^a,b^, There is a statistical difference between the averages shown with different letters (*p* < 0.05). SEM: Standard error of means, EIO: Edible inner organs, Pro: Proventriculus, L: Linear, Q: quadratic. ANOVA: analysis of variance.

**Table 5:** Meat color and pH of broiler with dietary supplemented sage leaf.

Parameters	Sage Leaf, g/kg				SEM	*p*-Value		
	0	S2	S4	S8		ANOVA	L	Q
Breast meat								
L*	50.92	50.52	52.55	52.24	0.39	0.187	0.087	0.952
a*	14.34	14.11	13.49	13.35	0.19	0.118	0.001	0.307
b*	14.00	13.38	14.14	13.84	0.17	0.405	0.854	0.639
pH	5.86	5.89	5.85	5.81	0.02	0.321	0.175	0.245
Thigh meat								
L*	55.88	55.96	56.83	57.77	0.425	0.410	0.072	0.586
a*	14.95	14.96	14.59	13.58	0.234	0.123	0.033	0.266
b*	15.06	14.61	15.36	15.70	0.187	0.232	0.070	0.118
pH	6.20	6.08	6.12	6.11	0.018	0.140	0.194	0.119

Values represent means of four replicate pens with 8 birds from each treatment; SEM Standard error of means. L*: lightness; 0 = black and 100 = white, a*: redness; −60 = green and 60 = red, b* yellowness; −60 blue and 60 = yellow, L: Linear, Q: quadratic. ANOVA: analysis of variance.

**Table 6:** Intestinal histomorphology of broiler with dietary supplementation sage leaf.

Parameters	Sage Leaf, g/kg				SEM	*p*-Value		
	0	S2	S4	S8		ANOVA	L	Q
Duodenum								
VL (μm)	1095.35	1128.01	1039.24	1145.02	19.25	0.224	0.724	0.340
CD (μm)	64.49 ^b^	67.30 ^b^	81.49 ^a^	71.96 ^b^	1.79	0.003	0.015	0.063
VL/CD	17.24 ^a^	17.25 ^a^	12.98 ^b^	16.47 ^a^	0.47	0.001	0.083	0.042
Jejunum								
VL (μm)	1175.52 ^a,b^	1267.70 ^a^	1105.95 ^b^	1278.49 ^a^	19.77	0.001	0.301	0.001
CD (μm)	78.71 ^b^	81.84 ^b^	83.06 ^b^	98.65 ^a^	1.64	0.001	0.001	0.035
VL/CD	15.04 ^a,b^	15.74 ^a^	13.64 ^c,b^	13.15 ^c^	0.28	0.001	0.001	0.278
Ileum								
VL (μm)	546.16 ^b^	789.27 ^a^	729.13 ^a^	561.82 ^b^	17.61	0.001	0.893	0.001
CD (μm)	66.22 ^a^	66.67 ^a^	50.23 ^b^	61.29 ^a,b^	2.28	0.021	0.005	0.006
VL/CD	8.57 ^b^	12.55 ^b^	14.53 ^a^	9.53 ^b^	0.98	0.001	0.073	0.001

Values represent means of four replicate pens with 8 birds from each treatment; ^a–c^, There is a statistical difference between the averages shown with different letters (*p* < 0.05). SEM: Standard error of means, VL: Villi length, CD: Crypt depth, VL/CD: Villi length/Crypt depth, L: Linear, Q: quadratic. ANOVA: analysis of variance.

**Table 7:** Cecum microbiota of broiler with dietary supplementation sage leaf (CFU g^−1^).

Parameters	Sage Leaf, g/kg				SEM	*p*-Value		
	0	S2	S4	S8		ANOVA	L	Q
*Enterococcus* spp.	7.00 ^a,b^	7.07 ^a^	6.41 ^c,b^	6.28 ^c^	0.13	0.033	0.003	0.371
*E. coli*	6.96	7.38	6.95	6.66	0.13	0.283	0.233	0.275
*Lactobacillus* spp.	7.49	7.66	7.61	7.94	0.09	0.396	0.138	0.672

Values represent means of four replicate pens with 8 birds from each treatment; ^a–c^, There is a statistical difference between the averages shown with different letters (*p* < 0.05). SEM: Standard error of means, CFU: Colony-forming units, L: Linear, Q: quadratic. ANOVA: analysis of variance.

**Table 8:** Effect of sage leaf supplementation on the shelf life of broiler breast meat (mg malondialdehyde/kg).

Parameters	Sage Leaf, g/kg				SEM	*p*-Value		
	0	S2	S4	S8		ANOVA	L	Q
3. day	0.14	0.13	0.13	0.11	0.009	0.795	0.340	0.899
5. day	0.12	0.13	0.13	0.11	0.011	0.595	0.466	0.741
7. day	0.19	0.14	0.17	0.13	0.011	0.157	0.180	0.773

Values represent means of four replicate pens with 8 birds from each treatment; L: Linear, Q: quadratic. ANOVA: analysis of variance.

## Data Availability

The original contributions presented in this study are included in the article. Further inquiries can be directed to the corresponding author.

## Abbreviations

The following abbreviations are used in this manuscript:

**Abbreviation**	**Description**
a*	Redness
b*	Yellowness
BWG	Body weight gain
CFU	Colony forming units
CP	Crude protein
EIO	Edible inner organs
FCR	Feed conversion ratio
FI	Feed intake
HCL	Hydrochloric acid
IW	Initial weight
L*	Lightness
LAB	Lactic acid bacteria
MDA	Malondialdehyde
ME	Metabolic energy
MEA	Malt extract agar
MRS	De Man Rogosa Sharpe agar
SEM	Standard error of means
TBA	Thiobarbituric acid
